# Differential methylation of G-protein coupled receptor signaling genes in gastrointestinal neuroendocrine tumors

**DOI:** 10.1038/s41598-021-91934-5

**Published:** 2021-06-10

**Authors:** Seyoun Byun, Kajsa E. Affolter, Angela K. Snow, Karen Curtin, Austin R. Cannon, Lisa A. Cannon-Albright, Ramya Thota, Deborah W. Neklason

**Affiliations:** 1 Huntsman Cancer Institute, University of Utah, 2000 Circle of Hope, Salt Lake City, UT 84112-5550 USA; 2 Department of Biomedical Informatics, University of Utah School of Medicine, Salt Lake City, USA; 3 Department of Pathology, University of Utah, Salt Lake City, USA; 4 Division of Epidemiology, Department of Internal Medicine, University of Utah, Salt Lake City, USA; 5 Division of General Surgery, Department of Surgery, University of Utah School of Medicine, Salt Lake City, USA; 6 Medical Oncology, Intermountain Healthcare, Salt Lake City, USA

**Keywords:** Prognostic markers, Gastrointestinal system, Cancer epigenetics

## Abstract

Neuroendocrine tumors (NETs) of the small intestine undergo large chromosomal and methylation changes. The objective of this study was to identify methylation differences in NETs and consider how the differentially methylated genes may impact patient survival. Genome-wide methylation and chromosomal copy number variation (CNV) of NETs from the small intestine and appendix were measured. Tumors were divided into three molecular subtypes according to CNV results: chromosome 18 loss (18LOH), Multiple CNV, and No CNV. Comparison of 18LOH tumors with MultiCNV and NoCNV tumors identified 901 differentially methylated genes. Genes from the G-protein coupled receptor (GPCR) pathways are statistically overrepresented in the differentially methylated genes. One of the highlighted genes from the GPCR pathway is somatostatin (*SST*), a clinical target for NETs. Patient survival based on low versus high methylation in all samples identified four significant genes (*p* < 0.05) *OR2S2*, *SMILR*, *RNU6-653P,* and *AC010543.1*. Within the 18LOH molecular subtype tumors, survival differences were identified in high versus low methylation of 24 genes. The most significant is *TRHR* (*p* < 0.01), a GPCR with multiple FDA-approved drugs. By separating NETs into different molecular subtypes based on chromosomal changes, we find that multiple GPCRs and their ligands appear to be regulated through methylation and correlated with survival. These results suggest opportunities for better treatment strategies for NETs based on molecular features.

## Introduction

Well-differentiated neuroendocrine tumors (NETs), previously referred to as carcinoids, can occur anywhere in the body, with the majority occurring in the gastrointestinal tract. Small intestinal NETs makeup almost half of these gastrointestinal NETs (GINETs) and are rare, with an incidence of 0.87 per 100,000 population per year^1–3^. Since GINETs are rare cancers, the molecular mechanisms driving pathologic changes remain elusive. Small intestinal NETs arise from enterochromaffin cells, which reside alongside the epithelial layer lining the lumen of the digestive tract, predominantly in the small intestine and appendix, where they regulate intestinal motility and secretion via the production of serotonin and other peptides^4,5^. Excess production of serotonin by the tumor may cause carcinoid syndrome, characterized by flushing and diarrhea, which occurs with about 17% of appendiceal and 32% of small intestinal NETs^6^.

GINETs often express neuroendocrine markers, such as synaptophysin, chromogranin, and somatostatin receptors (SSTRs) 1 to 5, which are G-protein coupled receptors and detected by immunohistochemistry. Positron emission tomography (PET) imaging using radiolabeled SSTR ligand tracers such as 68 Ga-DOTATE and 68 Ga-DOTATOC is a highly sensitive modality to diagnose patients with local or distant well-differentiated GINETs and also to evaluate for the potential role of somatostatin analogue (SSA) therapy (octreotide or lanreotide) in patients with metastatic NETs^7,8^. SSAs regulate hormonal hypersecretion, notably serotonin and other vasoactive substances in tumors expressing one or more subtypes of somatostatin receptors. Currently, metastatic GINETs are treated with the standard first-line SSA therapy, but there is evidence that different molecular subtypes are associated with different progression-free survival^9^. A better understanding of the molecular characteristics underlying GINETs may provide guidance for understanding the biology, the prognosis, and the selection of patients for more effective and targeted treatments.

The most frequent genomic alteration in small intestinal NETs is large chromosomal deletions. Most notably, full arms of chromosomes 18 are lost in 40–80% of tumors^10–12^. Inactivating mutations in *CDKN1B* are found in about 8% of small intestinal NETs, but small intestinal NETs otherwise are genetically stable, and somatic mutations in individual genes are uncommon^13,14^. Epigenetic modifications, in particular DNA methylation, have been proposed as a mechanism for small intestinal NET development^9^. DNA methylation can regulate gene expression and is an established mechanism for developing multiple types of cancer^15^. In general, but not exclusively, hypermethylation in promoter regions tends to decrease gene expression by blocking DNA binding sites for transcription factors^16–18^.

Multiple studies have considered the clinical significance of the loss of heterozygosity in chr18 (18LOH) with inconsistent results^19^. Karpathakis et al. suggested that small intestinal NETs with 18LOH are associated with favorable progression free survival following resection^9^. Yao et al. reported the 18LOH patients to have better survival outcomes^10^. Kim et al. found no significant difference in overall survival between those with and without 18LOH^20^. Although there is evidence of methylation changes in small intestinal NETs with 18LOH, the molecular pathways are not well defined^9^.

The objective of this study was to identify methylation differences in small intestinal and appendiceal NETs based on genomic alteration (molecular subtype) and to consider how the differentially methylated genes may impact survival.

## Materials and methods

All methods were carried out in accordance with relevant guidelines and regulations. The study was approved by the University of Utah Institutional Review Board. Informed consent was obtained from all living participants.

### Research participants

Potential NET patient cases including both small intestine and appendix (histology codes 8240, 8241, 8243, 8244, 8246; ICDO locations 170, 171, 172, 173, 178, 179, 181) in Utah between 1999 and 2014 were identified through the Utah Cancer Registry (UCR). UCR is a Surveillance Epidemiology and End Results (SEER) registry that has collected all cancers diagnosed in Utah from 1966 to the present. Cases from subjects under 18 years old and in situ cases were excluded, leaving 552 potential NET subjects for study (Table 1). Additional cases with a diagnosis from 2014 to 2018 were identified through University of Utah Health electronic medical records and referred to the study by the Department of Surgery. UCR referred 77 cases reported to UCR by the University of Utah Health and had a pathology number on record. The Department of Surgery referred 12 additional recent cases. From these 89 cases, 47 subjects had archived FFPE blocks available, were confirmed as a well-differentiated neuroendocrine tumor by study pathologist (KA), and had sufficient tissue for methylation analysis. Confirmation by the study pathologist was essential as multiple cases were not assigned the correct histology code.

**Table 1:** Characteristics of NET cases tested.

	Utah cancer registry cases excluding those tested	Cases tested	Molecular classification by copy number variation (CNV)		
			18LOH	NoCNV	MultiCNV
Number of cases	516	47	19	20	8
Diagnosis years	1999–2014	1999–2018	1999–2016	2003–2018	2011–2018
Race Caucasian	500 (97%)	44 (94%)	18 (94%)	18 (90%)	8 (100%)
Male	285 (55%)	28 (60%)	10 (52%)	12 (60%)	6 (75%)
Average age at diagnosis (range)	62.1 (19–90)	60.6 (25–87)	59.8 (44–87)	59.2 (25–77)	66.0 (50–85)
**Tumor location [ICD-O-3 site codes]**					
Small intestine [170–173, 178, 179]	475 (92%)	44 (94%)	19 (100%)	17 (85%)	8 (100%)
Appendix [181]	41 (8%)	3 (6%)	0	3	0
**Stage [SEER summary stage]**					
Localized [1]	170 (33%)	12 (26%)	1 (5%)	8 (40%)	3 (38%)
Regional [2, 3, 4]	224 (43%)	23 (49%)	11 (58%)	9 (45%)	3 (38%)
Distant [7]	110 (21%)	12 (26%)	7 (37%)	3 (15%)	2 (25%)
Unknown [9]	12 (2%)	0	0	0	0
**Grade**					
Total tumors graded	224 (43%)	37 (79%)	17 (89%)	12 (60%)	8 (100%)
Grade 1	155 (69%)	26 (70%)	12 (71%)	9 (70%)	5 (63%)
Grade 2	59 (26%)	10 (27%)	5 (29%)	3 (25%)	2 (25%)
Grade 3	10 (4%)	1 (3%)	0	0	1 (13%)
Unknown grade	292 (57%)	10 (21%)	2 (11%)	8 (40%)	0
Tobacco use indication	159 (31%)	13 (28%)	7 (37%)	4 (20%)	2 (25%)
Family history of small intestinal NET	14 (3%)	1 (2%)	1 (5%)	0	0

### Methylation analysis of tumor DNA

Formalin-fixed paraffin-embedded (FFPE) blocks were serially cut in 5 μm increments and mounted onto charged slides. Hematoxylin and eosin (H&E) staining of a representative slide was used to identify areas of neuroendocrine tumor (> 50% tumor) by the study pathologist (KA). Cells were microdissected from the marked tumor region, and DNA was extracted from FFPE tissue slides using a QIAamp DNA FFPE Tissue Kit [Qiagen #56404] using the manufacturer's protocol with the following modifications: (1) paraffin was removed from the slides prior to microdissection, and the tissue was added directly to Buffer ATL (2) there were two 56 °C incubations with Proteinase K: overnight, and then for an hour after supplementing with an additional 20 µL Proteinase K (3) for the elution, 2 × 25 µL of ATE was added to the center of the membrane and incubated for 5 min at room temperature before eluting.

Following extraction, DNA concentrations were measured with a Qubit™ dsDNA BR assay kit [ThermoFisher Scientific #Q32850 & #Q32856]. Bisulfite conversion was performed on 250 ng DNA per sample using a Zymo EZ DNA Methylation Kit [Zymo Research #D5001] following the manufacturer's recommended protocol for methylation microarrays. DNA quality was evaluated with an Infinium FFPE QC kit [Illumina #WG-321-1001] prior to performing the recommended FFPE DNA Restore protocol [Illumina #WG-321-1002 & Zymo #D4024]. MethylationEPIC BeadChip microarrays [Illumina #WG-317-1002] were used for genome-wide methylation profiling. The microarrays were processed with an Illumina iScan platform using the Infinium HD Methylation assay protocol with the recommended modifications for FFPE samples. The raw methylation IDAT data was analyzed using GenomeStudio (RRID:SCR_010973) v2011.1 software with Methylation Module v1.9 [Illumina].

### DNA methylation data pre-processing

The methylation data were pre-processed following a pipeline of the ChAMP (RRID:SCR_012891) R package (V.2.13.5)^21^. Probes were filtered out using the *champ.filter* function with the following criteria: probes with (1) detection *p* > 0.01, (2) less than three beads in at least 5% of samples, (3) non-CpG cytosine, (4) polymorphic nucleotide, (5) multiple regions of the genome and (6) chromosome X or Y. Total of the 699,602 CpG sites were selected for further analysis. After the quality control, the type-II probe bias was corrected by the method of beta mixture quantile (BMIQ) normalization according to pipeline defaults.

### Copy number variants using methylation data

**Figure. 1:**
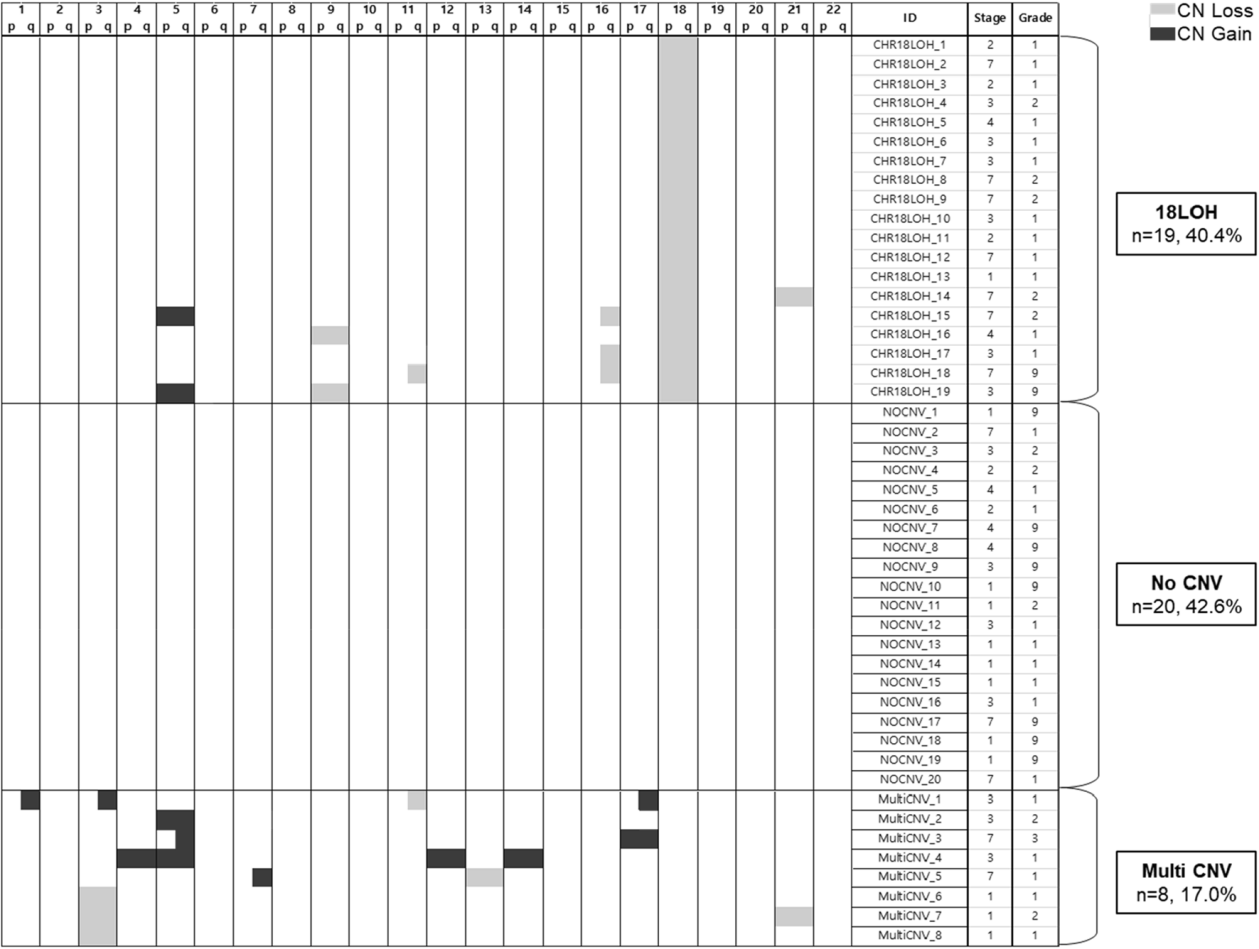
Chromosomal rearrangements (molecular subtypes) of neuroendocrine tumors. The ChAMP result referring copy number variation, including loss and gain of copy number. Samples were grouped into three subgroups: loss of chromosome 18 (18LOH), no copy number variation (NoCNV) or multiple copy number variations (MultiCNV). Black indicates gain of copy number. Grey indicates loss of copy number.

In order to identify copy number variation (CNV) in our 47 NET cases, intensity values for each probe were used to count copy numbers and determine if alterations were present. Copy number of our NETs were compared with previously published Illumina MethylationEPIC BeadChip data measured from ten normal myometrial samples in NCBI GEO database (GSM3417135-GSM3417144)^22^. CNV was estimated and compared from the intensity value using the *champ.CNA* function of ChAMP^23^. The whole-chromosome arm CNV was defined with the criteria of length > 80% and segment mean >  ± 0.2. The 47 NETs were classified into three groups according to CNV results: Chromosome 18 loss of heterozygosity (18LOH), no chromosome arm copy number alterations (NoCNV), and multiple copy number alterations excluding those with 18LOH (MultiCNV) (Fig. 1).

### Differential methylation analysis

The averaged methylation value (β-value) of all CpGs within the promoter region of a gene was compared between the three CNV groups (i.e., 18LOH vs*.* NoCNV, 18LOH vs. MultiCNV, and MultiCNV vs. NoCNV) with an unpaired Wilcoxon's test to identify differentially methylated genes. A parallel analysis of singular CpG sites that are differentially methylated was done (Supplementary Table S2). The promoter region was considered 1500 bp upstream and 200 bp downstream from the transcription start site (TSS). A total of 39,252 genes were tested. An FDR adjusted *p* value below 0.05 and change in methylation greater than 15% (Δβ > 0.15) were used to identify genes with statistically significant differential methylation levels. The pheatmap package in R was used to create the heatmap. The default method for “Euclidean” distance and the heatmap clustering method Ward.D was used (v 1.0.12)^24^.

### Functional enrichment and network analysis

Functional enrichment analysis using ConsensusPathDB (RRID:SCR_002231) (Release 33, CPDB; http://cpdb.molgen.mpg.de/)^25^ was used to interpret the functional role of genes identified with differential methylation status. Significant pathways and GO terms were defined as having an adjusted q-value < 0.05 (*p* value corrected for multiple testing based on the number of pathways used). To reduce redundancy and remove potential false-positive GO terms, we used the GO-module web-tool (http://lussierlab.org/GO-Module) (v.1.3)^26^. Protein–protein interaction (PPI) network was constructed using StringDB V.11 (STRING, RRID:SCR_005223, http://string-db.org)^27^ with an interaction combined score of > 0.9, which represents the highest confidence score of the various evidence (i.e., text mining, database, and co-expression). PPI network was visualized by using Cytoscape (RRID:SCR_003032) v3.5.1^28^.

### Survival analysis

Survival in months after diagnosis was determined from death certificates (n = 16). If the individual was alive at last contact (n = 31), a study-end month was set at June 2019, the most recent contact date among research subjects.

Overall survival outcome was evaluated using Kaplan–Meier survival analysis with several comparing criteria. Analyses were conducted according to the three molecular subgroups (i.e., 18LOH, NoCNV, and MultiCNV). We also considered survival based on the methylation status of gene promoters (n = 39,252 genes including coding, noncoding, and pseudogenes). The 47 NET samples were separated into two groups according to β-value (i.e., low- and high-methylated groups) using k-means clustering with the number of clusters set at two for survival analysis. Additionally, within the 18LOH tumors (n = 19) group, survival was compared based on high versus low methylation according to the β-value using k-means clustering with the number of clusters set at two. We defined statistically significant associations as a *p* value < 0.05.

## Results

### Overview of the study cohort

Although the number of tumors tested for methylation (1999–2018) was modest, this subset was generally representative of all small intestine and appendix NET cases reported to the UCR in 1999–2014 (Table 1). None of the differences in the values reported in Table 1 reach statistical significance. In those cases tested, a larger percentage had tumor grade information available: 79% versus 43% for the cases who did not undergo testing. This is expected because the cases tested, on average, were more recent diagnoses and came from an academic institution where tumor grading was more common. Restricting the comparison to tumors that were graded, the tumors tested versus tumors of cases that were not tested were similar in the frequency of grade, with the majority (70% and 69%, respectively) being grade 1. Known risk factors of tobacco use and family history were similar between cases with and without testing as well. Survival did not significantly differ between the two groups (*p* = 0.76; Supplementary Fig. S1).

### Genome-wide DNA methylation copy number variation profiling

Analysis of copy number variation (CNV) in the tumor, based on DNA methylation array data, showed losses on the entire chromosome 18 (18LOH) in 19 out of 47 tumors (40.4%) and gain of chromosome 5 in 5 tumors (10.6%), similar to previous reports^9,10,19,20^. Multiple CNV (MultiCNV) were observed in 8 tumors (17.0%), and no copy-number alternation (NoCNV) was observed in the remaining 20 tumors (42.6%), three of which are from the appendix (NoCNV_6, NoCNV_7, NoCNV_8; Fig. 1). No statistically significant differences in patient demographics or tumor characteristics were observed, but this was limited by the small numbers (Table 1). Tumors with 18LOH trend towards a more advanced stage and higher rates of tobacco use than NoCNV or MultiCNV tumors, but this difference is not significant.

### Differential DNA methylation analysis in the three subgroups

**Figure 2:**
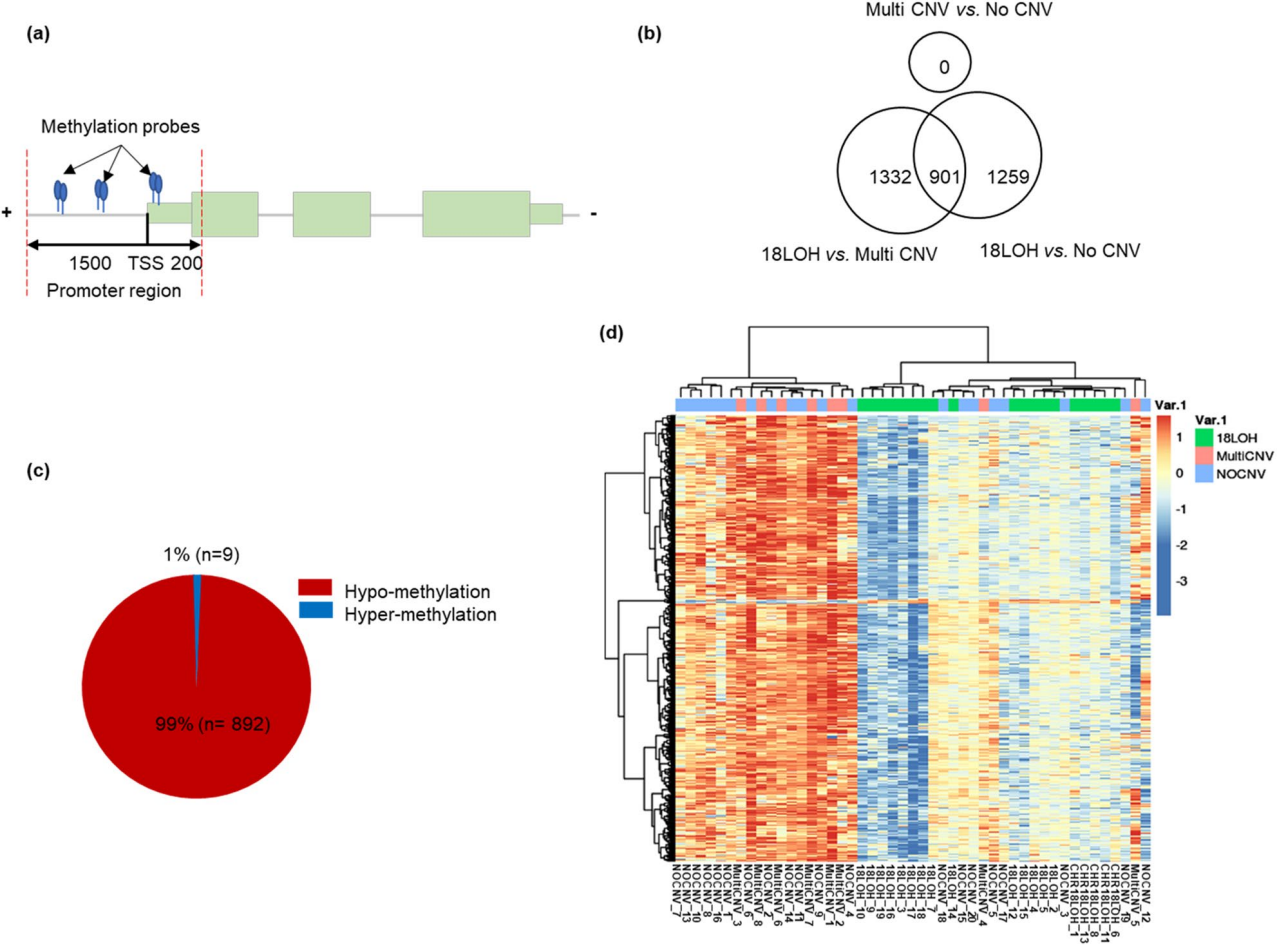
Differential methylation status between molecular subtypes. (**a**) A schema methylation probes within promoter region. Methylation probes between − 1500 and 200 bp from the transcript start site are considered. (**b**) The number of significantly differential methylation genes of each comparison. The overlapped differential methylation of 18LOH versus Multi CNV and 18LOH versus No CNV is considered as 901 genes specific to 18LOH. (**c**) Proportion of hypomethylated (n = 892, 99%) and hypermethylated (n = 9, 1%) genes in 18LOH tumors. (**d**) Heatmap of 901 differentially methylated genes for each tumor. Blue indicates hypomethylation. Red indicates hypermethylation. Tumor molecular subtypes are indicated in green (18LOH), pink (MultiCNV) or blue (NoCNV).

A subset of 196,354 CpG methylation sites out of a total of 699,602 CpG sites was mapped to specific promoters (39,252 genes) for analysis (Fig. 2a). Differential methylation was examined in individuals with a history of tobacco use versus those without. Hierarchical clustering of differentially methylated genes showed no difference between tumors of those who have a history of tobacco use versus those that do not. To identify differentially methylated genes related to 18LOH, intergroup comparisons were performed for 18LOH *vs*. MultiCNV, 18LOH *vs*. NoCNV, and MultiCNV *vs*. NoCNV (FDR < 0.05 and Δβ > 0.15); the number of differentially methylated genes are 1332, 1259, and 0, respectively when considering average promoter methylation (Fig. 2b) and 6790, 8923, and 0 respectively when considering singular CpG methylation (data not shown). An intersection of differentially methylated genes in the 18LOH group was identified; 901 genes when average promoter methylation is used and 2230 genes (2945 singular CpGs) when singular CpG methylation is used (Supplementary Table 2). The 901-gene set for average differential promoter methylation was examined further, but not the singular CpG analysis because it can have issues with independence and false positives^29^. Interestingly, none of the 901 genes with differential methylation reside on chromosome 18 (Supplementary Table 1) but do reside on the other 21 autosomal chromosomes. Aside from having a reduced intensity, the β-values for methylation probes on chromosome 18 in the 18LOH tumors are normal. We hypothesize that the lack of differentially regulated genes on chromosome 18 is due to hypomethylation preceding and promoting 18LOH, and once chromosome 18 is lost, methylation differences in the remaining chromosome are not observed. Another interesting observation is that 205 (22.8%) of the 901 differentially methylated genes were annotated as long noncoding RNA (lncRNA). Heatmap clustering of the 901 genes shows that these genes are predominately hypomethylated in 18LOH tumors when compared to MultiCNV and NoCNV groups. (Fig. 2c). Only 9 genes (1%) were hypermethylated in the 18LOH tumors *(SST, DMBT1, RNU6-1039P, RP11-37N22.1, Lnc-FAM241A-1, RP11-153K16.1, CERS3-AS1, RP11-542M13.3*, and *AC025278.2*; Fig. 2c). Hierarchical clustering of the tumors based on methylation of the 901 genes shows the separation of the 18LOH tumors from the other two molecular subtypes (Fig. 2d). The three neuroendocrine tumors from the appendix which were included in the study cluster with the NoCNV molecular subtype (NoCNV_6, NoCNV_7, NoCNV_8). When the analysis was repeated, removing these three appendiceal samples, very similar results were found. The coefficient of correlation was 0.962 when comparing the *p* value for methylation differences of the 901 genes from the original analysis of 47 tumors to the analysis of 44 tumors excluding appendiceal NETs (Supplementary Fig. 5).

### Functional enrichment analysis and network of significant methylation of 18LOH tumors

**Figure 3:**
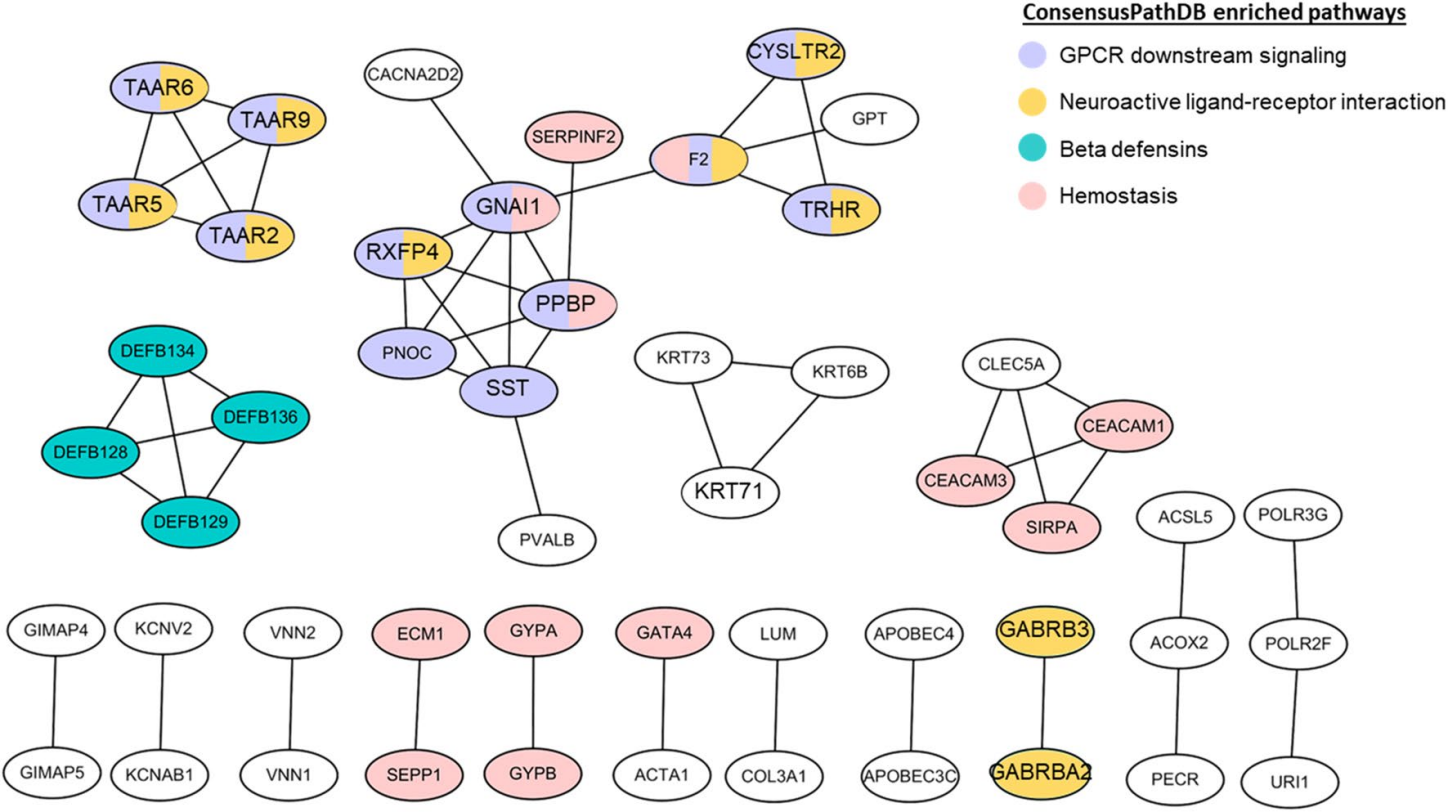
Protein–protein interaction network of 901 genes with differential methylation in 18LOH group. STRING Database with input of the 901 genes predicted protein–protein interactions which are either direct (physical) or indirect (functional). Genes that met the highest confidence for interaction (> 0.9) are shown in the Fig. (51 genes). Lines connect genes that were predicted for interacting. Overlay of pathways that are overrepresented in the set of 901 genes, identified four pathways in this subset of 51 genes. Genes assigned to the pathway are indicated as follows: GPCR signaling (purple); Neuroactive ligand-receptor interaction (yellow); Beta-defensins (green) and Hemostasis (pink). For genes assigned to multiple pathways, the circle is multiple colors.

Overrepresentation analysis of the 901 genes, using ConsensusPathDB, revealed 12 enriched functional pathways (q-value < 0.05) (Supplementary Fig. S2). This includes multiple signaling-related pathways: GPCR signaling (including olfactory receptors), defensin and beta-defensin, neuroactive ligand-receptor interaction, vitamin D receptor, and hemostasis. Within the set of 901 differentially methylated genes, 18 Gene Ontology (GO) pathways were significantly over-represented (q-value < 0.05), including “Olfactory receptor activity” (GO:0004984), “Sensory perception of chemical stimulus” (GO:0007606), “G-protein-coupled receptor signaling pathway” (GO:0004930), “defense response to bacterium” (GO:0042742), and “humoral immune response” (GO:0006959) (Supplementary Fig. S2). The protein–protein interaction (PPI) network constructed with the STRING database identified 51 genes with direct interactions (Fig. 3). When genes from the 12 enriched functional pathways were superimposed on the PPI network, the 51 genes were functionally grouped into four subgroup pathways: GPCR downstream signaling (including olfactory receptors), neuroactive ligand-receptor interaction, beta-defensins, and hemostasis. The GPCR downstream signaling pathway is highly related to cancer progression behaviors such as proliferation, angiogenesis, and metastasis^30–32^. Multiple genes were assigned to both GPCR and neuroactive ligand-receptor interaction pathways.

When the 47 tumors were grouped into high methylation versus low methylation for all 39,252 genes and not considering their CNV status, four genes were significantly associated with survival based on methylation status, *p* < 0.05; hypermethylation confers better survival for *OR2S2*, *RNU6-653P* and *AC010543.1* and hypomethylation confers better survival for *SMILR* (Supplementary Fig. S4). Conflicting reports on survival of patients with SINETs with chromosome 18 loss led us to also consider heterogeneity in methylation within this group. The 19 tumors with 18LOH were separated into high methylation or low methylation for each of the 901 differentially methylated genes, and survival analysis was compared. A significant difference in survival (*p* < 0.05) was observed for 24 of the genes (Supplementary Table S1). Two are included in the PPI network (Fig. 3): *TRHR* (*p* = 0.006) and *VNN2* (*p* = 0.050).

**Figure 4:**
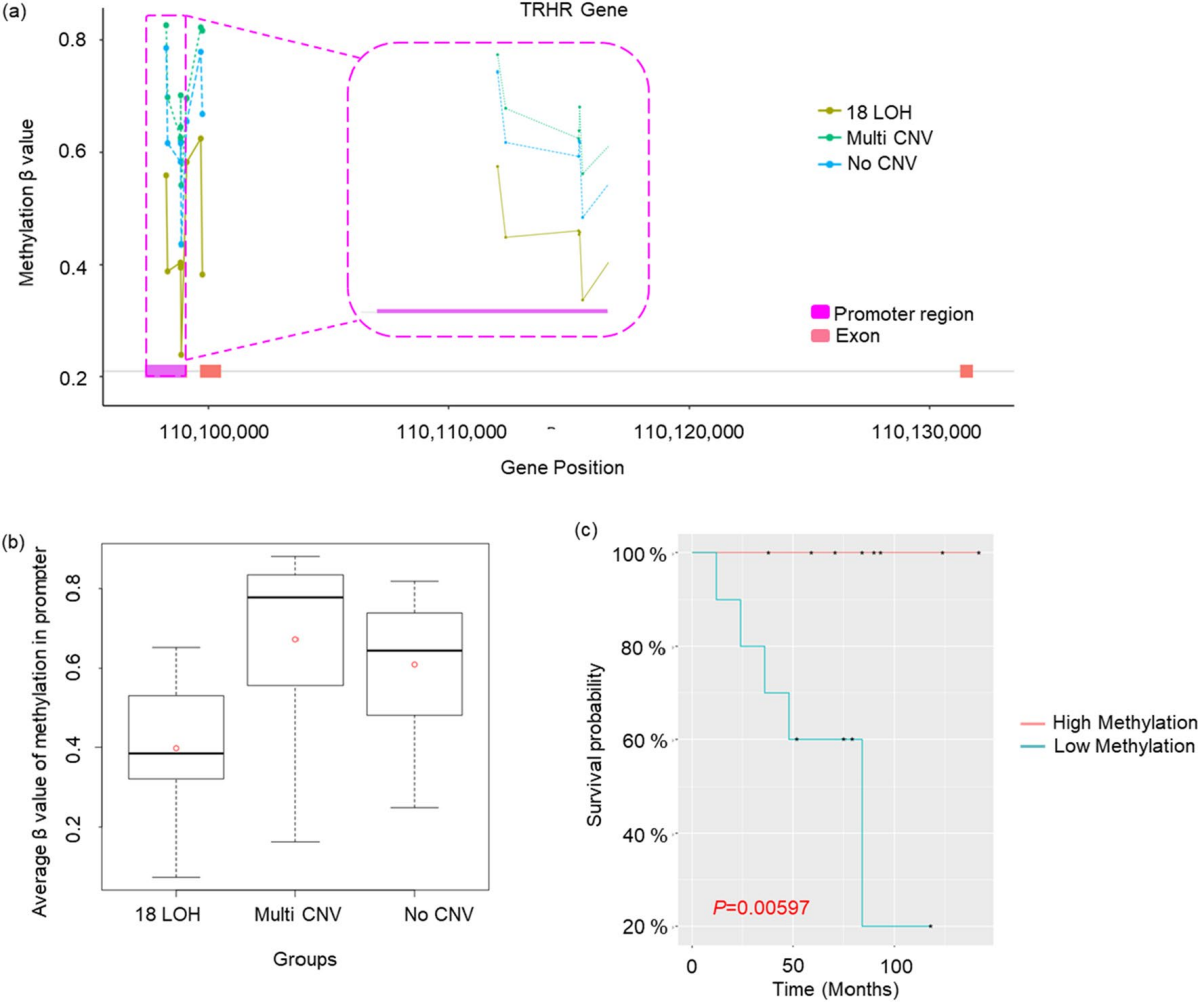
Methylation status of *TRHR* gene promoter. (**a**) A plot of beta-values of methylation probes within the promoter (Y-axis) and genomic coordinates (X-axis). The promoter region (pink), exon (orange) and intron are presented a bottom line of the graph. (**b**) A boxplot including distributions of average beta-value of methylation probes in the promoter for each molecular subtype. (**c**) Survival analysis comparing 18LOH tumors with high methylation (red line; samples 18LOH_2, _4, _5, _6, _7, _9, _14, _15, _16) and low methylation (blue line; samples 18LOH_1, _3, _8, _10, _11, _12, _13, _17, _18, _19) of *TRHR* promoter.

By way of example, two genes whose methylation may have biological importance in neuroendocrine tumors are included in the large protein–protein interaction network in Fig. 3: *TRHR* (thyrotropin-releasing hormone receptor), a G-protein coupled receptor, and *SST* (somatostatin), a ligand to the *SSTR* G-protein coupled receptor. The *TRHR* promoter is hypomethylated on all six CpG methylation sites in tumors with 18LOH relative to tumors with NoCNV (*p* = 0.004) or with MultiCNV (*p* = 0.04) (Fig. 4a,b. Survival analysis of the 18LOH tumors suggests that low methylation of the *TRHR* gene confers poor survival *p* = 0.005 (Fig. 4c). Methylation of the TRHR promotor silences transcription of the gene in thyroid cancers^33^. This suggests that reduced *TRHR* expression due to promoter methylation improves survival.

**Figure 5:**
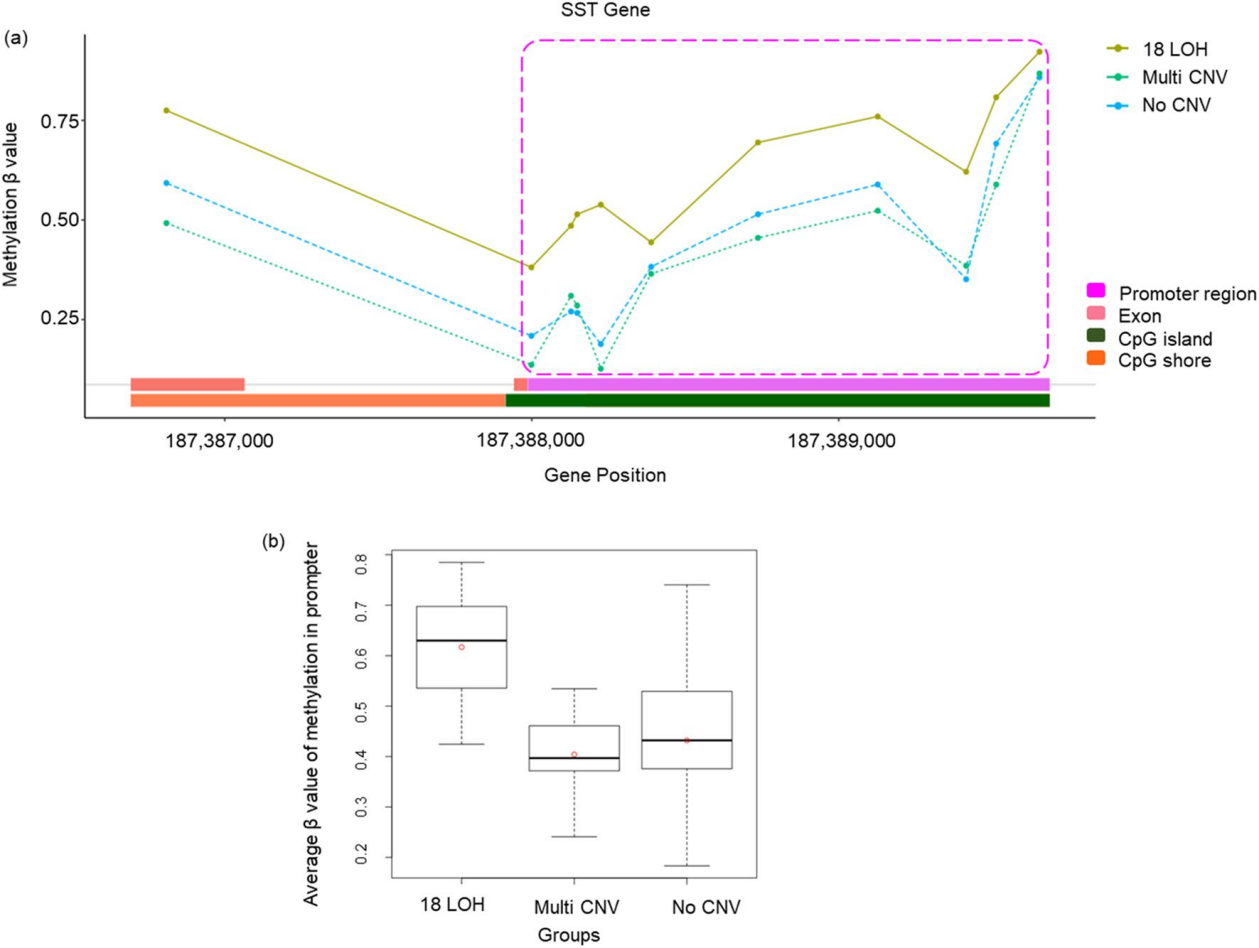
Methylation status in the *SST* gene promoter. (**a**) A plot of beta-values of methylation probes within the promoter (Y-axis) and genomic coordinates (X-axis). The promoter region (pink), exon (orange), intron, CpG island (green), and shore (dark orange) are presented a bottom line of the graph. (**b**) A boxplot including distributions of average beta-value of methylation probes in the promoter for each molecular subtype.

The *SST* promoter is hypermethylated in the 18LOH tumors relative to the MultiCNV (*p* = 0.006) and NoCNV (*p* = 0.002) tumors (Fig. 5a,b). Hypermethylation of the *SST* promoter in gastric cancers results in decreased mRNA and protein expression, thereby reducing somatostatin's ability to inhibit tumor growth^34^. The somatostatin protein binds to G-protein coupled somatostatin receptors, and analogs of somatostatin are a common treatment for GINETs^8^.

## Discussion

In this study, we investigated differential DNA methylation status in well-differentiated small intestinal and appendiceal NETs with different molecular subtypes. We found 901 genes with differentially methylated promoter regions in the study's 18LOH NETs, and these genes were enriched in tumor-related pathways, including “GPCR downstream signaling”, “neuroactive ligand-receptor interaction”, “beta-defensins”, and “hemostasis”. Most of the genes were hypomethylated, broadly suggesting transcriptional activation of these genes. None of the 901 genes resided on chromosome 18, suggesting that hypomethylation of chromosome 18 may contribute to its loss in NETs. Chromosomal instability in tumors is promoted by DNA hypomethylation and is observed in multiple tumor types including pancreatic NETs^35,36^. The lncRNA, which made up 22.8% of the differentially methylated genes, has been shown to regulate cancer genes and represent additional disease pathways^37–40^. We examined the influence of tobacco use on methylation in GINET tumors. However, differences were not observed when comparing tumors from patients with and without an indication of tobacco use^41^. This may be due to the small number of samples, timing of use (current versus past), and/or the quantity (pack-years)^42^.

The GPCR pathway represents a large family of cell-surface molecules regulating the signal transmission for multiple cellular functions. GPCRs are also the most common class of therapeutic targets, with approximately 700 FDA-approved drugs targeting 128 GPCRs^43,44^. Pertinent to the findings of this study, GPCRs play multiple roles in cancer development and have been the focus of past studies to define differences in neuroendocrine tumors, primarily those originating from the small intestine versus pancreas and lung^45,46^. Our significantly enriched GPCR pathways suggest that expression of GPCR pathways is controlled through methylation events, which drives tumor progression a subset of GINETs^46,47^. Being neuroendocrine tumors, it makes sense that the “neuroactive ligand-receptor interaction” pathway is involved, and accordingly, is part of the GPCR downstream signaling pathway. We describe hypermethylation of one such neuroactive ligand, the *SST* gene encoding somatostatin, in 18LOH tumors relative to NoCNV and MultiCNV. Somatostatin is the ligand for SSTRs, G-protein coupled somatostatin receptors that are overexpressed in a subset of GINETs and the target of somatostatin analog therapies. Examining the clinical response to somatostatin analog therapies relative to *SST* methylation state may provide guidance on more targeted treatment strategies.

Our survival analysis with the three molecular subgroups based on chromosomal changes showed there was no significant difference between subgroups (*p* = 0.41; Supplementary Fig. S3). However, the ability to detect such differences is limited by the sample size. Survival analysis based on promoter methylation did identify four associated genes *OR2S2, SMILR*, *RNU6-653P,* and *AC010543,* although very little is known about their biologic function, and nothing is published related to cancer. We also identified 24 genes with methylation levels associated with survival outcomes, specifically in 18LOH tumors, potentially explaining why there is disagreement in the literature on survival outcomes for 18LOH tumors. The most significant association was with *TRHR.* Hypomethylation of the *TRHR* promoter may be associated with 18LOH tumor progression and survival outcomes. The mechanism behind this survival difference is not obvious but may represent a constellation of methylation events that are clinically relevant, potentially as a pharmacologic target for small intestinal NET patients.

Selection of tumors from the cancer registry included ICDO codes for the small intestine as well as the appendix because we believe there is cross-over in the etiology of these tumors. Three of the 47 tumors examined for methylation were from the appendix. All three were classified as NoCNV subtype, and hierarchical clustering of their methylation profile was fully integrated with the other NoCNV samples from the small intestine, suggesting a similar etiology. Additionally, we have observed neuroendocrine tumors of the appendix in families with multiple NETs of the small intestine, which suggests there is overlap in the genetic etiology.

Limitations of the study included a small sample size, methylation arrays that were not run on matched normal tissues, and a lack of treatment data for the NET patients. Also, the methylation changes were not validated with RNA expression profiling but rather relied on published results in different tissues and frequently observed (but not exclusive) negative correlation between promoter methylation and gene expression in other studies^18^. This study lays the foundation for future work to address these limitations. Strengths are that the samples tested were representative of all tumors reported in the state for that period of time. The frequency of molecular subtypes was similar to previous reports^9,11–13^.

In conclusion, by separating small intestine and appendiceal NETs into different molecular subtypes based on chromosomal changes, we were able to define major pathways that are differentially methylated. Two relevant pathways were the GPCR and the overlapping neuroactive ligand-receptor interaction. One gene that is differentially hypermethylated in 18LOH tumors is *SST*, encoding somatostatin. Somatostatin analogs are a primary treatment for GINETs, targeting overexpressed somatostatin receptors on the tumors. Somatostatins are also used for GINET visualization, radiotherapy, and repressing carcinoid-syndrome side effects. GPCR may be important for survival in small intestinal NETs and are targetable by drugs in some cases. In future work, methylation status in these genes can be explored as a prognostic and/or predictive biomarker to predict responses to SSA's and for careful selection of patients for appropriate treatments. Future studies that incorporate detailed histologic characterization, GPCR expression, and treatments will be important. The molecular characterization of GINETs can lead to novel prognostic and predictive biomarkers to better inform treatments and may improve the survival of patients with GINETs.

## Supplementary Information

Supplementary Information 1.

Supplementary Information 2.

Supplementary Information 3.
