# Supplementary material for: Differential methylation of G-protein coupled receptor signaling genes in gastrointestinal neuroendocrine tumors
Source: Sci Rep. 2021 Jun 10;11:12303. doi: 10.1038/s41598-021-91934-5 (PMC8192774; doi:10.1038/s41598-021-91934-5)
Supplement: Supplementary file 1 — Supplementary Information 1. [file 41598_2021_91934_MOESM1_ESM.pptx]

## Slide 1
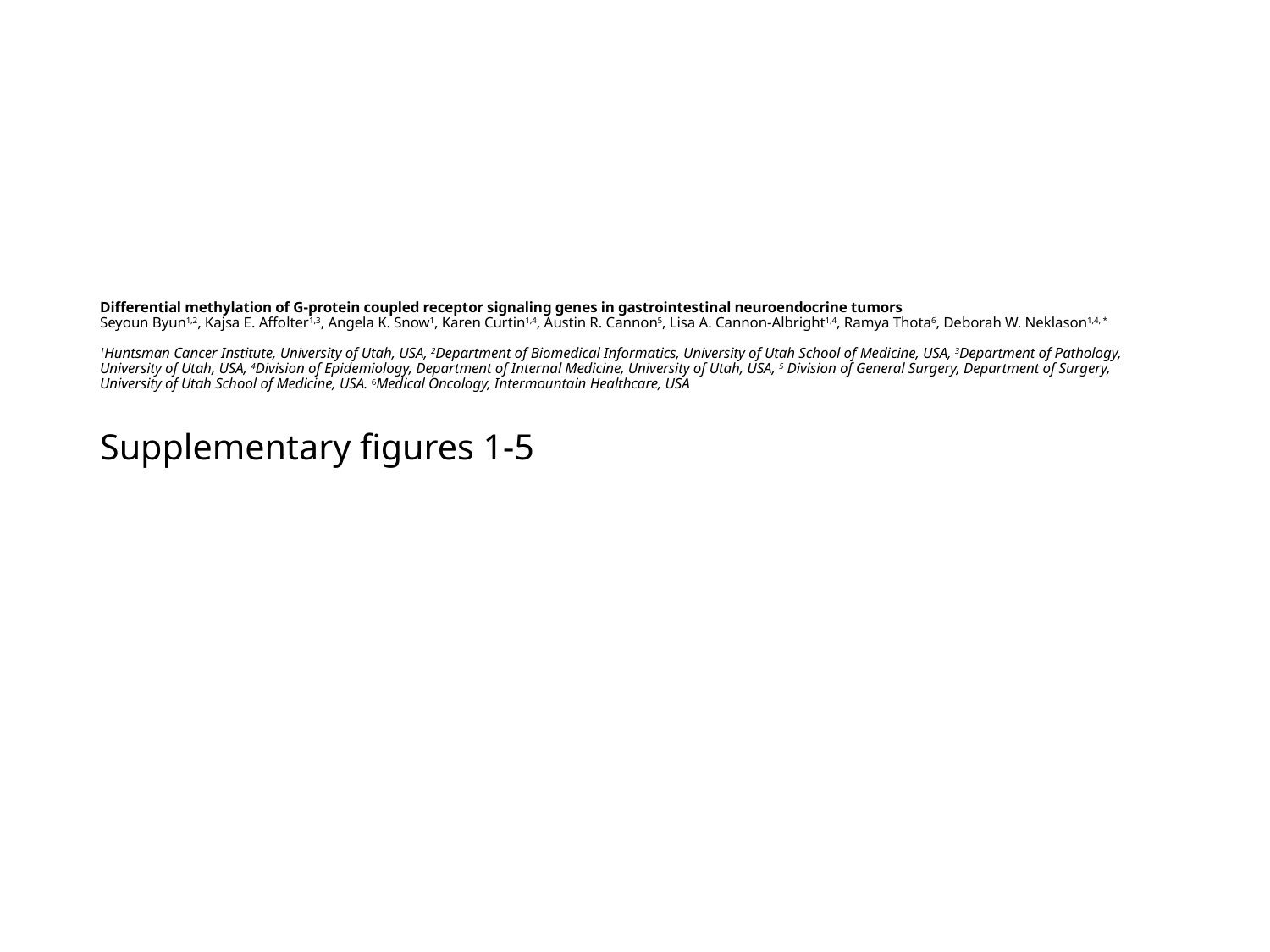

# Differential methylation of G-protein coupled receptor signaling genes in gastrointestinal neuroendocrine tumors Seyoun Byun1,2, Kajsa E. Affolter1,3, Angela K. Snow1, Karen Curtin1,4, Austin R. Cannon5, Lisa A. Cannon-Albright1,4, Ramya Thota6, Deborah W. Neklason1,4, * 1Huntsman Cancer Institute, University of Utah, USA, 2Department of Biomedical Informatics, University of Utah School of Medicine, USA, 3Department of Pathology, University of Utah, USA, 4Division of Epidemiology, Department of Internal Medicine, University of Utah, USA, 5 Division of General Surgery, Department of Surgery, University of Utah School of Medicine, USA. 6Medical Oncology, Intermountain Healthcare, USA Supplementary figures 1-5

## Slide 2
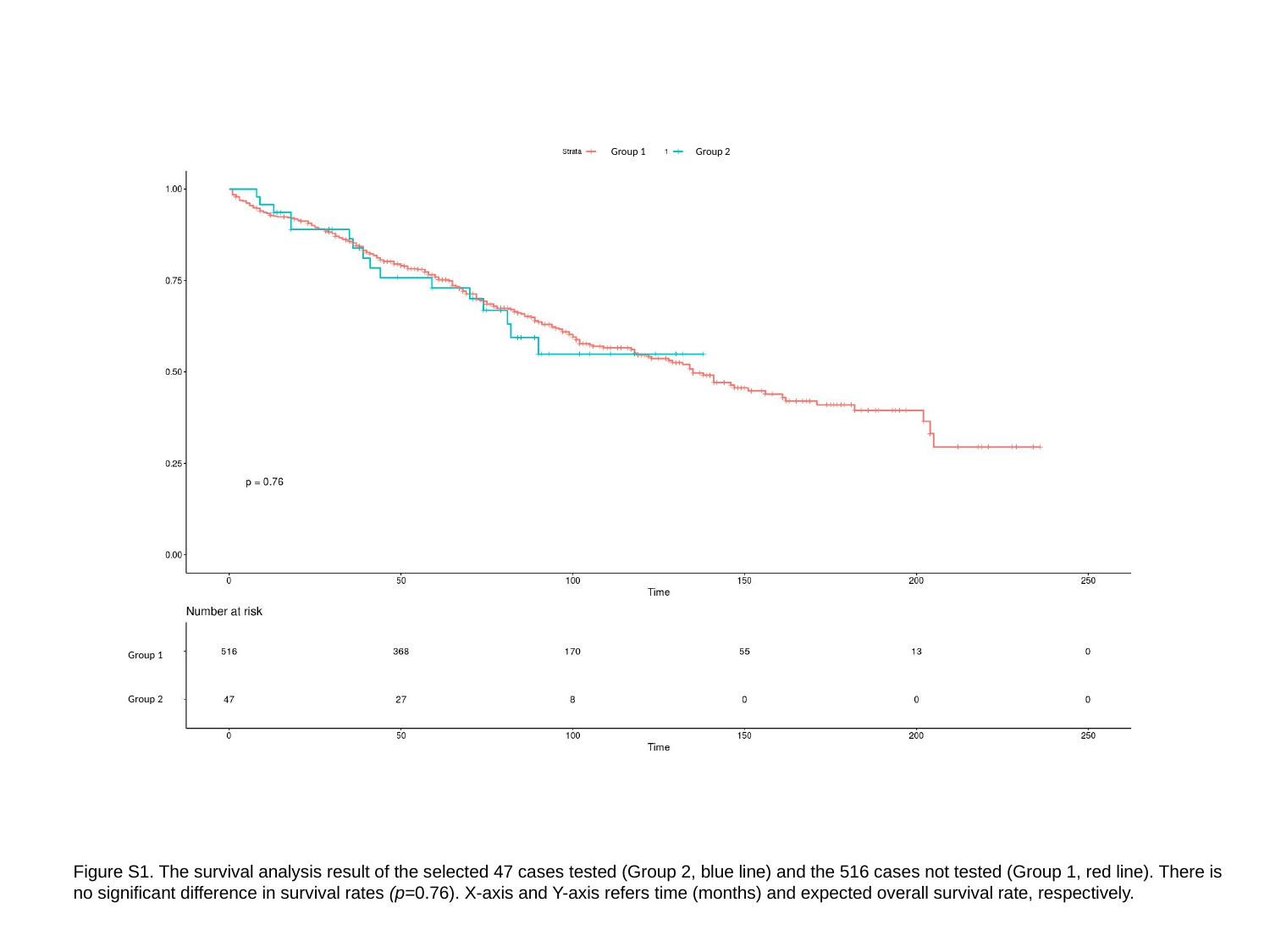

Group 1
Group 2
Group 1
Group 2
Figure S1. The survival analysis result of the selected 47 cases tested (Group 2, blue line) and the 516 cases not tested (Group 1, red line). There is no significant difference in survival rates (p=0.76). X-axis and Y-axis refers time (months) and expected overall survival rate, respectively.

## Slide 3
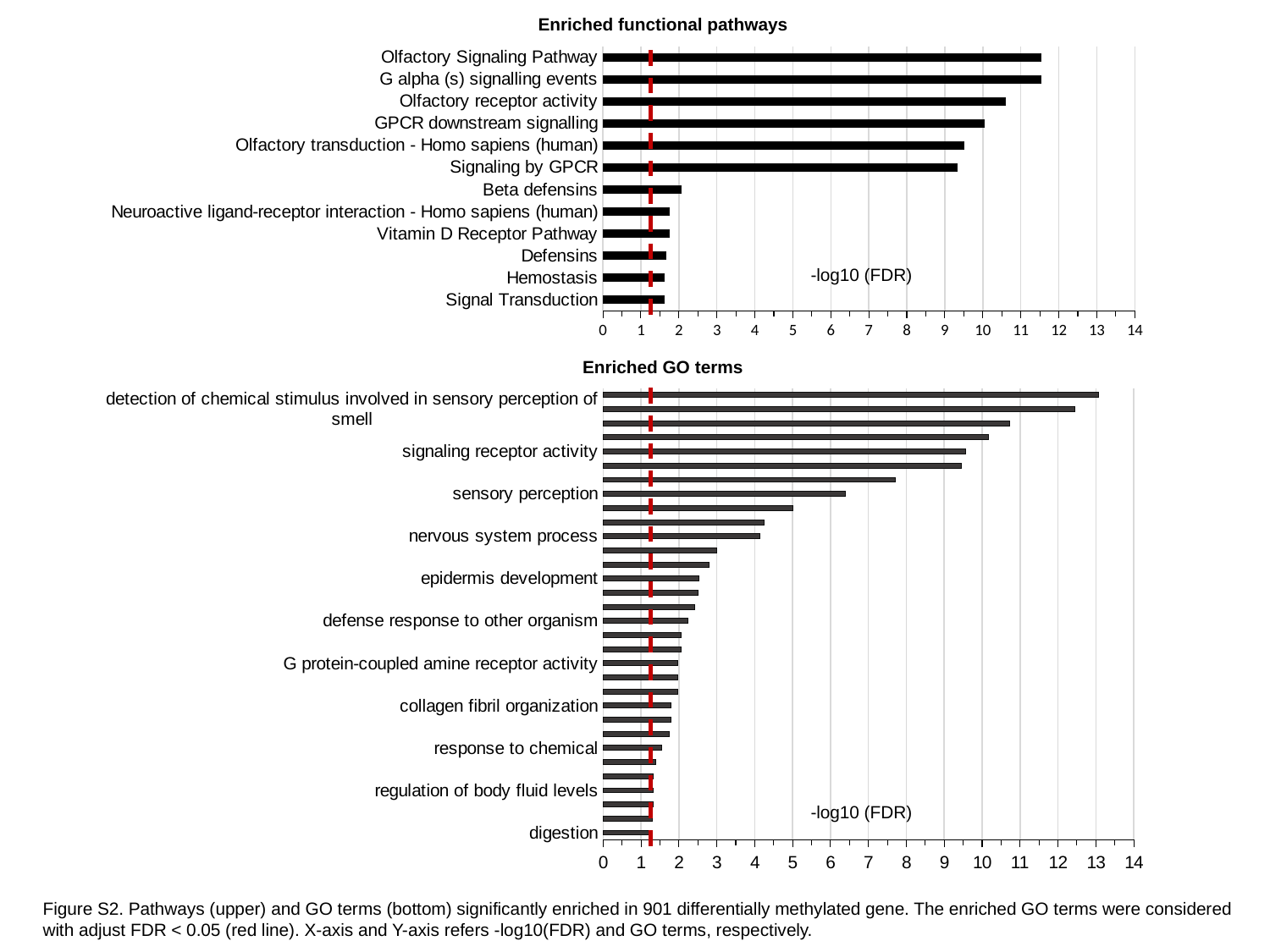

Enriched functional pathways
### Chart
| Category | |
|---|---|
| Signal Transduction | 1.6130859873031398 |
| Hemostasis | 1.6179112237309172 |
| Defensins | 1.6689354073232183 |
| Vitamin D Receptor Pathway | 1.7446244955391277 |
| Neuroactive ligand-receptor interaction - Homo sapiens (human) | 1.7446244955391277 |
| Beta defensins | 2.05003665366813 |
| Signaling by GPCR | 9.33023536094208 |
| Olfactory transduction - Homo sapiens (human) | 9.507964862427578 |
| GPCR downstream signalling | 10.030950653316546 |
| Olfactory receptor activity | 10.587675409926792 |
| G alpha (s) signalling events | 11.52895808693994 |
| Olfactory Signaling Pathway | 11.52895808693994 |-log10 (FDR)
Enriched GO terms
### Chart
| Category | |
|---|---|
| digestion | 1.3024494016954604 |
| killing of cells of other organism | 1.3024494016954604 |
| blood coagulation | 1.3207457220799963 |
| regulation of body fluid levels | 1.3207457220799963 |
| humoral immune response | 1.3207457220799963 |
| response to bacterium | 1.4004712926526657 |
| response to chemical | 1.5360075717938915 |
| organ growth | 1.737041936385987 |
| defense response to bacterium | 1.7892727363277812 |
| collagen fibril organization | 1.7892727363277812 |
| natriuretic peptide receptor activity | 1.97424964457848 |
| pantetheine hydrolase activity | 1.977367525777241 |
| G protein-coupled amine receptor activity | 1.977367525777241 |
| response to external biotic stimulus | 2.052081254633168 |
| coagulation | 2.0691431548880383 |
| defense response to other organism | 2.2314774112226834 |
| response to other organism | 2.4091317496933464 |
| response to biotic stimulus | 2.5067904437641215 |
| epidermis development | 2.5239398605613643 |
| epidermal cell differentiation | 2.7919083769146584 |
| skin development | 2.9902372912501507 |
| nervous system process | 4.126361205175493 |
| keratinization | 4.24425205745337 |
| system process | 5.004501630311566 |
| sensory perception | 6.4032335020082805 |
| G protein-coupled receptor signaling pathway | 7.703721080198935 |
| transmembrane signaling receptor activity | 9.45024249496398 |
| signaling receptor activity | 9.560276996365069 |
| G protein-coupled receptor activity | 10.177574265346449 |
| sensory perception of chemical stimulus | 10.735968355875105 |
| detection of chemical stimulus involved in sensory perception of smell | 12.434386762144001 |
| olfactory receptor activity | 13.062775692194608 |-log10 (FDR)
Figure S2. Pathways (upper) and GO terms (bottom) significantly enriched in 901 differentially methylated gene. The enriched GO terms were considered with adjust FDR < 0.05 (red line). X-axis and Y-axis refers -log10(FDR) and GO terms, respectively.

## Slide 4
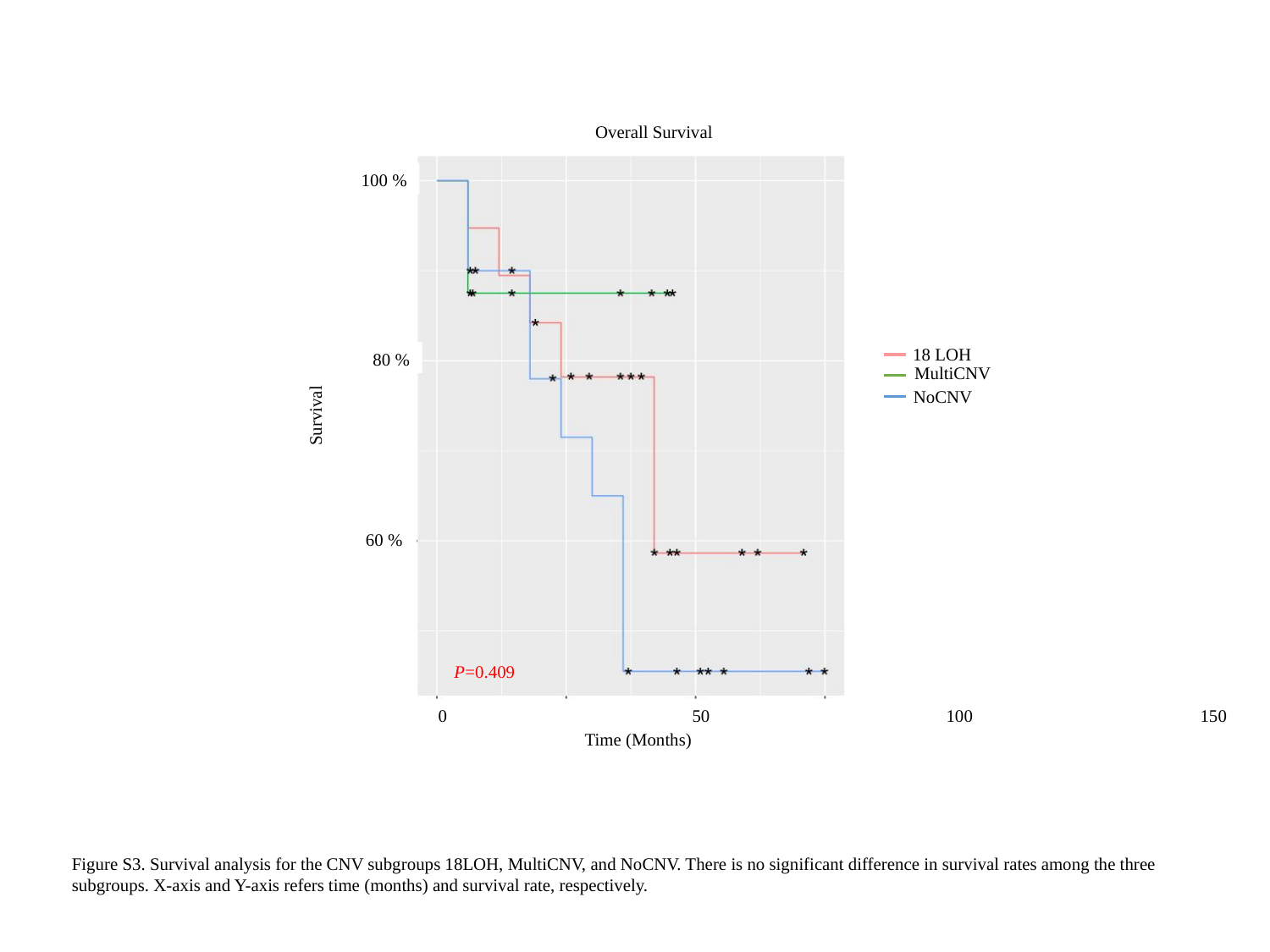

Overall Survival
100 %
18 LOH
80 %
MultiCNV
NoCNV
Survival
60 %
P=0.409
0		50		100	 	150
Time (Months)
Figure S3. Survival analysis for the CNV subgroups 18LOH, MultiCNV, and NoCNV. There is no significant difference in survival rates among the three subgroups. X-axis and Y-axis refers time (months) and survival rate, respectively.

## Slide 5
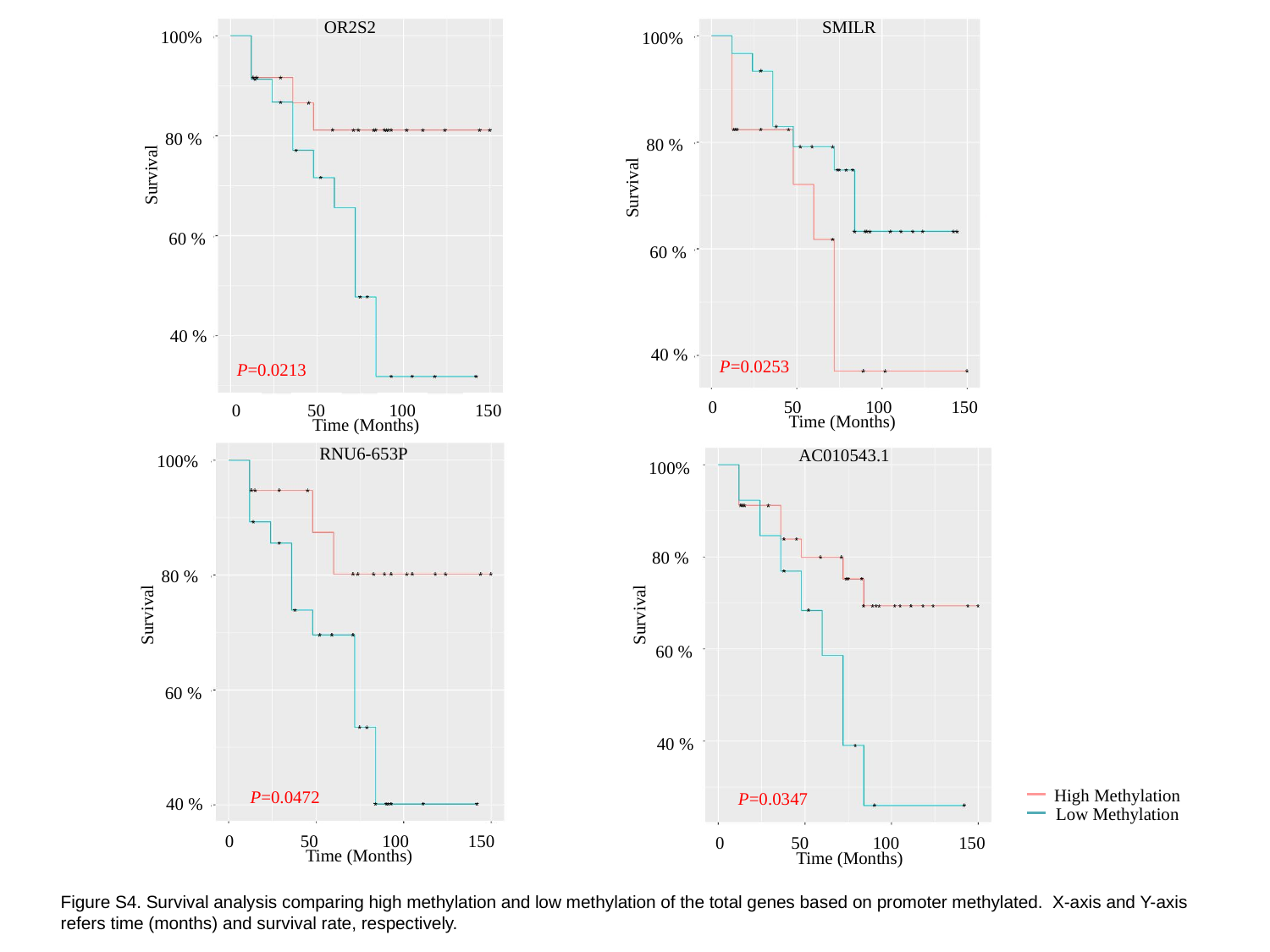

OR2S2
SMILR
P=0.0253
100%
100%
80 %
80 %
Survival
Survival
60 %
60 %
40 %
40 %
P=0.0213
0
50
100
150
0
50
100
150
Time (Months)
Time (Months)
RNU6-653P
AC010543.1
100%
100%
80 %
80 %
Survival
Survival
60 %
60 %
40 %
High Methylation
P=0.0472
P=0.0347
40 %
Low Methylation
0
50
100
150
0
50
100
150
Time (Months)
Time (Months)
Figure S4. Survival analysis comparing high methylation and low methylation of the total genes based on promoter methylated. X-axis and Y-axis refers time (months) and survival rate, respectively.

## Slide 6
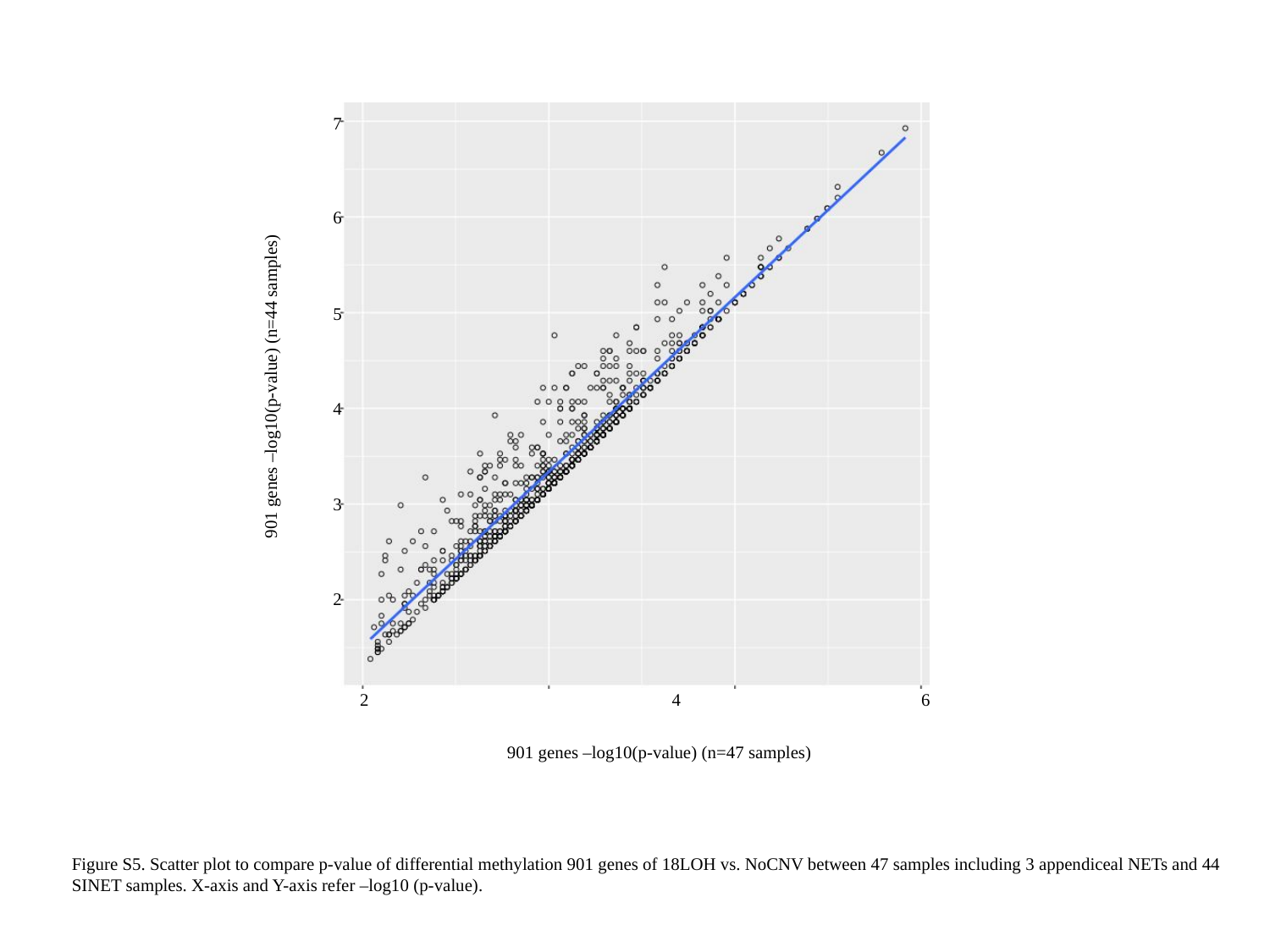

7
6
901 genes –log10(p-value) (n=44 samples)
5
4
3
2
2		 4		 6			 8
901 genes –log10(p-value) (n=47 samples)
Figure S5. Scatter plot to compare p-value of differential methylation 901 genes of 18LOH vs. NoCNV between 47 samples including 3 appendiceal NETs and 44 SINET samples. X-axis and Y-axis refer –log10 (p-value).
